# The Influence of Diet on Tinnitus Severity: Results of a Large-Scale, Online Survey

**DOI:** 10.3390/nu14245356

**Published:** 2022-12-16

**Authors:** Steven C. Marcrum, Milena Engelke, Hazel Goedhart, Berthold Langguth, Winfried Schlee, Markku Vesala, Jorge P. Simoes

**Affiliations:** 1 Department of Otolaryngology, University Hospital Regensburg, 93053 Regensburg, Germany; 2 Department of Psychiatry and Psychotherapy, University of Regensburg, Bezirksklinikum Regensburg, 93053 Regensburg, Germany; 3 TinnitusHub, London WC2H 9JQ, UK; 4 Institute for Information and Process Management, Eastern University of Applied Sciences, 9000 St. Gallen, Switzerland

**Keywords:** tinnitus, nutrition, diet, caffeine, alcohol, salt, online survey, mHealth

## Abstract

Optimization of dietary intake is an essential component in the multidimensional effort to prevent and manage chronic disease. Recently, demand has increased for nutrition-focused management strategies for chronic tinnitus. The primary aim of this study was to evaluate 10 dietary items for their association with changes in subjective tinnitus severity. A secondary aim was to develop an algorithm to better identify those individuals who might benefit from dietary modification strategies. A total of 5017 anonymous users of the TinnitusTalk forum completed an online survey regarding how various dietary items affected the severity of their tinnitus. Results suggest that, while intake of caffeine [positive effect (PE): 0.4%; negative effect (NE): 16.2%], alcohol (PE: 2.7%; NE: 13.3%, and salt (PE: 0.1%; NE: 9.9%) was most likely to influence tinnitus severity, it did so only for a small proportion of participants and reported effects were most commonly mild. Further, though a classification algorithm was able to leverage participant demographic, comorbidity, and tinnitus characteristics to identify those individuals most likely to benefit from dietary modification above chance levels, further efforts are required to achieve significant clinical utility. Taken together, these results do not support dietary modification as a primary treatment strategy for chronic tinnitus in the general population, though clinically meaningful effects might be observable in certain individuals.

## 1. Introduction

Tinnitus, or the conscious awareness of a tonal or composite noise for which there is no identifiable external sound source [1], is estimated to affect between 10–15% of the general population, though prevalence rates of 30% and more have been reported for certain demographic groups [2,3,4]. For approximately one-fifth of those affected, quality of life is sufficiently impaired that clinical intervention is needed [5]. However, even for those not requiring professional attention, persistent exposure to tinnitus can negatively influence mood, concentration, memory, and sleep quality, among other effects [6]. While there is unlikely to be a singular cause of tinnitus, converging evidence suggests that it arises from an up-regulation of sensory gain and a concomitant degradation of inhibition across various structures along the auditory pathway, most commonly in conjunction with peripheral auditory system damage [7,8,9]. Unfortunately, there is currently neither a cure nor a widely accepted protocol for the management of tinnitus. Rather, a wide variety of treatment strategies are available, usually approaching tinnitus from medical, psychological, audiological, and holistic perspectives [10,11,12]. In recent years, however, demand has increased for nutrition-focused management of chronic tinnitus [13].

Three general approaches can be applied to the investigation of nutrition’s effects on specific health outcomes, as follows: single nutrient analysis, single food analysis, and dietary pattern analysis [14]. While single nutrient methods have the potential to be more robust against confounds than single food- and dietary pattern-based methods, they might also obscure the complex effects of individual foods or foods in combination, a factor of great significance for individuals attempting to make practical decisions about what to eat. Dietary pattern-based methods, on the other hand, better account for the effects of various foods in combination and might deliver more valid evidence for the association between nutrition and disease. However, much as for single nutrient methods, the individual contribution of single foods remains unclear, and recommendations based on such methods might be difficult for end consumers to implement. Single food analysis can be viewed as a compromise between the other two methods, being both less specific and less comprehensive than single nutrient and dietary pattern analysis, respectively; however, such an approach has the significant advantage of generating recommendations that are simple, intuitive, and easy to integrate into everyday life. Thus, single food recommendations are also the type most commonly requested and discussed by everyday people.

Optimization of nutrient intake, alongside regular physical activity and lifestyle modification, is an essential component in the multidimensional effort to prevent and manage chronic disease [15,16]. Though identification of a causal relationship between specific dietary factors and disease outcomes is often impaired by our incomplete understanding of nutrient physiology, the complexity of interactions between diet and individual patient characteristics, such as comorbidities, and the slow-developing nature of many chronic diseases, it is nonetheless accepted that diet significantly influences outcomes in most chronic conditions, including cardiometabolic disease [17,18,19], site-specific cancer [20,21,22,23], osteoporosis [24,25], and disabling dementia [26,27]. Increasingly, investigations have begun reporting insights into the interplay between nutrition and chronic pathology of the auditory system [28,29,30,31], with some results suggesting implications for forestalling presbycusis, increasing resilience against the deleterious effects of noise, and reducing the incidence or severity of tinnitus symptoms [29,32,33,34,35]. Despite progress, however, optimal nutrient sources, doses, and combinations for the promotion of hearing health have yet to be established.

Specific recommendations arising from investigations into the influence of dietary factors on hearing and tinnitus outcomes vary (for review, see Spankovich and Le Prell [36]; Spankovich [37]), though higher intake of lipids, carbohydrates, and sugars are generally associated with poorer outcomes and diets more consistent with published dietary guidelines are associated with superior audiometric thresholds and reduced odds of persistent tinnitus. For example, in a longitudinal study of 3135 women, Curhan, Halpin [33] reported that those maintaining healthful dietary patterns (high in fiber and protein, low in sodium and saturated fat) exhibited a 25–30% lower risk of mid- and high-frequency hearing threshold elevation after 3 years, when compared with women following less healthful diets. Similarly, Dawes, Cruickshanks [29] found increased intake of vitamin D and diets characterized by higher intake of fruit, vegetables, and protein to be associated with decreased odds of hearing difficulties, while increased fat intake was associated with increased odds of hearing difficulties. Further, higher vitamin B12 and protein intake were associated with reduced odds of developing tinnitus. In a study of dietary fiber intake in 1194 adults over 50 years of age, Tang, Tran [38] reported low fruit-fiber (<3.6 g/day) and low cereal-fiber (<4.2 g/day) consumption to be associated with a 65% and 54% increase in the risk of developing tinnitus over the next 10 years, respectively. While the precise mechanisms of these effects are not yet clear, several authors have speculated that superior dietary nutrient intake might positively affect the auditory system by improving cochlear blood flow, lessening oxidative damage, and reducing inflammation [28,29,33,37].

Though the body of evidence suggesting that healthful eating might be of benefit in terms of hearing and tinnitus outcomes is slowly accumulating, few high-quality trials have been conducted to date, and reported effects have generally been weak. Furthermore, concerning tinnitus, the majority of studies have focused on the risk of developing tinnitus, whereas, for patients with chronic tinnitus, searching for nutrition-based treatment recommendations to lessen already present tinnitus symptoms is desired. In a recent systematic review evaluating the effects of caffeine consumption on tinnitus, Aljuaid, Mirza [39] concluded that increased caffeine intake might reduce the risk of developing tinnitus in persons without tinnitus; however, in persons with preexisting tinnitus, a reduction in caffeine consumption could be beneficial. Thus, the findings of studies focusing on the risk of developing tinnitus might be limited in terms of generalizability to those persons with preexisting tinnitus. Finally, methodological decisions in previous studies have resulted in participant groups not necessarily reflective of the wide variety of tinnitus experiences. Fortunately, recent technological advances in the area of crowdsensing are beginning to overcome this limitation by efficiently opening studies to inclusion of a worldwide, internet-based cohort. Indeed, crowdsensing is already being leveraged for a broad range of health-related applications [40,41,42,43], including some focused specifically on hearing loss and tinnitus [42,44,45]. Initial data indicate that, through the inclusion of large, nonclinical participant groups, more representative datasets can be collected, which might allow for the generation of more valid and clinically useful treatment recommendations [46].

While previous investigations have identified statistically significant relationships between various nutrients, foods, or dietary patterns and tinnitus-related outcomes, the general weakness of reported effects and inconsistencies in reported effects across studies and across participants within given studies represents an important limitation to actionable, evidence-based recommendations [47]. The primary aim of this study was, therefore, to extend existing knowledge of the association between key dietary items and tinnitus severity through the implementation of a large-scale, online questionnaire. A secondary aim was to develop an algorithm to better identify those individuals who might benefit from a dietary modification strategy. Through the use of multivariable analyses for the mapping of participant characteristics to reported effects of dietary items on tinnitus severity, we hoped to simultaneously increase classification accuracy and provide a candidate explanation for apparent contradictions between available reports using univariable analysis methods.

## 2. Materials and Methods

### 2.1. Participants

A total of 5017 users of an international, online forum for people with tinnitus, TinnitusTalk (www.tinnitustalk.com), anonymously participated in a 2016 survey on their tinnitus experience. Participants consented to having their data anonymously stored and analyzed for scientific purposes prior to completing the survey. As no personally identifiable information was collected and retrospective identification of individual participants was not possible, the requirement for ethical approval was waived by the Institutional Review Board of the University of Regensburg.

### 2.2. Procedure

Participants responded to survey questions pertaining to subjective tinnitus perception and severity (e.g., loudness, pitch, laterality, duration, etc.), comorbidities, and the effectiveness of previous tinnitus treatments [11]. In addition, participants subjectively rated the effect of a set of predetermined dietary items on their tinnitus perception. These effects were rated on a Likert scale ranging from 1 to 4, where 1 encoded ‘this food makes my tinnitus a lot worse’, 2 encoded ‘this food makes my tinnitus a little worse’, 3 encoded ‘this food makes my tinnitus a little better’, and 4 encoded ‘this food makes my tinnitus a lot better’. If applicable, users could also indicate that a given food item had no effect on their tinnitus. Ratings were obtained for 10 dietary items reported in the literature and among people with tinnitus to commonly be associated with a change in tinnitus perception. Specifically, ratings were obtained for alcohol, caffeine, chocolate, citrus-containing food, fatty food and/or junk food, monosodium glutamate (MSG), red meat, salt, spicy food, and sugar.

### 2.3. Data Preparation

Initial inspection of study results revealed systematic class imbalances (see Supplemental Figure S1). For example, participants were significantly more likely to select ‘’no effect’’ than any other option for all dietary items, and no participant reported chocolate, MSG, or salt as greatly improving tinnitus. Imbalance in datasets is a well-known problem in data mining, which serves to decrease the accuracy of machine learning algorithm outputs [48]. To minimize class imbalances, response categories 1 and 2 and categories 3 and 4 were combined, thereby representing overall positive and negative associations between tinnitus severity and a given dietary item, respectively. Participants were subsequently assigned to one of three groups, as follows: (1) participants whose tinnitus was negatively associated with any of the 10 dietary items, (2) participants whose tinnitus was positively associated with any of the 10 dietary items, and (3) participants whose tinnitus was not associated with any of the 10 dietary items. However, as classes between these three groups remained imbalanced after initial binning, participants were further grouped into the following groups: (1) participants whose tinnitus was affected by any of the 10 dietary items (either positive or negative association) and (2) participants whose tinnitus was not affected by any of the 10 dietary items. The resulting variable, which encoded whether changes in tinnitus perception were associated with intake of any of the dietary items assessed, was used as a dependent variable for binary classification.

**Figure 1:**
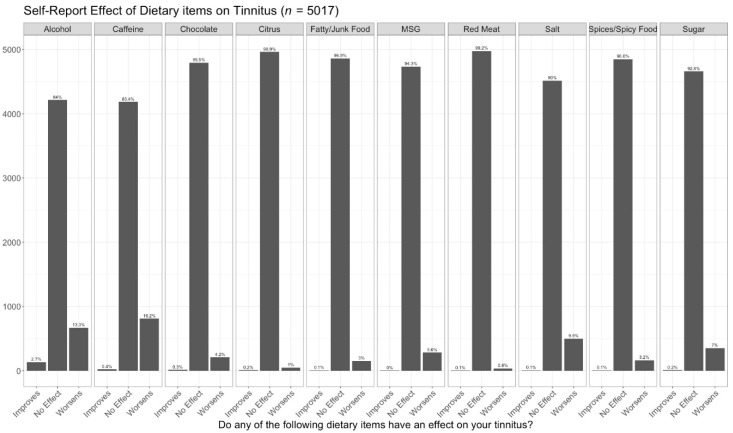
Reported associations of 10 dietary items with tinnitus.

After inspecting missing value patterns, we accepted the assumption that data were missing at random and imputed the missing data with the k-nearest neighbor algorithm [49]. All categorical variables were then converted to dummy variables, and predictors with near-zero variance were excluded from the analysis. Finally, levels of categorical variables representing less than 10% were grouped together to improve the model’s accuracy [50]. The synthetic minority over-sampling technique (SMOTE) was used to compensate for class imbalances along the dependent variable [51]. This technique has the benefit of generating new synthetic instances of the less-occurring classes (in our case dietary items that have either a positive or negative association with tinnitus, see Figure 1) to improve the performance of machine learning algorithms performing classification tasks.

### 2.4. Statistical Analysis

The data was split into training and testing datasets using an 80/20 ratio—that is, the model was tuned using 80% of the data (*n* = 4017) and tested on the remaining 20% of the data (*n* = 1307).

Covariates, such as sociodemographic factors, tinnitus characteristics, and reported comorbidities (Table 1), were used to predict whether participants’ tinnitus was influenced by dietary items using a random forest model. The random forest algorithm is an all-purpose algorithm proposed by Breinman [52], which has found widespread use in regression and classification tasks. A 10-fold cross-validation strategy from the training sample was used to tune the model’s hyperparameters with a grid-search strategy; specifically, mtry (i.e., the number of predictors to sample each split) and min_n (i.e., the number of observations necessary for splitting nodes). After the best performing model, defined as the model with the largest area under the curve (AUC), was identified in the training dataset, a last fit was performed in the testing dataset. Variable importance was assessed using the VIP package [53].

In the present analysis, categorical variables were reported as count (%), and the χ^2^ test was used to compare the frequencies of the variables conditioned on whether tinnitus was reported as being influenced by intake of dietary items. Two-sided *p*-values smaller than 0.05 were considered statistically significant, and only values corrected for multiple comparisons using the Holm method are reported [54]. Cramer’s V, which ranges from 0 to 1, was used as a measure of effect size and can be interpreted as analogous to coefficients from the Pearson’s correlation [55].

All analyses were conducted in R 4.2.1, [56] with the packages tidyverse [57] and tidymodels [58].

## 3. Results

Results summarizing participant demographics, reported comorbidities, and tinnitus characteristics, stratified according to whether any dietary item was reported as having affected tinnitus, are presented in Table 1. Of the 10 factors assessed, all but 3 indicated a statistically significant difference in terms of the observed frequency between the 2 groups (effect versus no effect). Thus, these findings suggest that neither gender, a pulsatile tinnitus characteristic, nor the presence of hearing loss are likely to be of benefit in determining the probability of a dietary item influencing tinnitus perception in isolation; however, participant age, the duration of tinnitus, the effect of noise on tinnitus, and tinnitus frequency, among others, might be. Nonetheless, it remains that no pattern consistent with current tinnitus theory could be identified across all factors and, despite achieving statistical significance even after correcting for multiple comparisons, all observed effect sizes were quite small (ranging from Cramer’s V = 0.02 to 0.18). Thus, the statistical significance of these factors might be better interpreted as a product of the survey’s large sample size, as opposed to the intrinsic importance of any individual factor.

Figure 1 displays the proportion of respondents reporting either a positive, negative, or neutral association with tinnitus severity for each of the 10 dietary items. Although the dietary items included in this study have all been suggested to affect tinnitus, none of them influenced tinnitus perception, either positively or negatively, for a majority of participants. Specifically, *no effect* was selected between 83.4–99.2% of the time across all dietary items. Interestingly, alcohol, the dietary item most commonly reported to improve tinnitus symptoms (2.7%) was simultaneously the second most likely to worsen symptoms (13.3%). Taken together, present results suggest non-neutral associations with dietary items are most likely to be negative, with the most pronounced negative associations being for caffeine (16.2%) and salt (9.9%). While none of the dietary items included in this study were effective in modulating tinnitus severity for a large proportion of participants, given the high prevalence of tinnitus and assuming the appropriateness of generalizing these results to larger populations, the proportions of affected participants observed in this study represent millions of people with tinnitus. Thus, the potential clinical significance of these findings nonetheless warrants further investigation.

As the dietary items investigated in this study were only influential for a subset of participants, we next assessed the performance of a random forest algorithm in terms of predicting whether dietary items would be associated with changes in tinnitus perception for each given participant. The algorithm used each individual’s demographic, comorbidity, and tinnitus characteristics (see Table 1) as inputs for the classification task. To mitigate class imbalances (see Table 1), participants whose tinnitus was either positively or negatively modulated by dietary items were binned together and classified against participants whose tinnitus was not modulated by dietary items.

**Figure 2:**
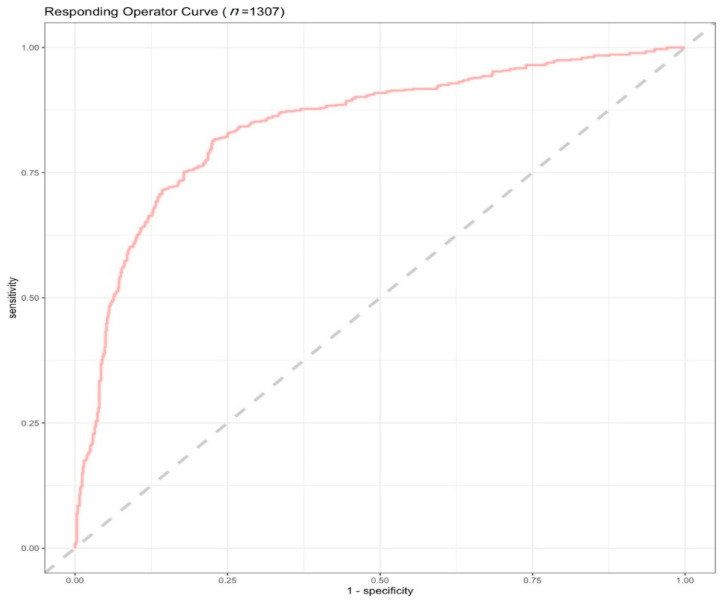
ROC curve of the final model in the test sample. A final AUC of 0.84 was observed.

Using 80% of the dataset (*n* = 4017), the algorithm was trained with a cross-validation strategy to identify optimal hyperparameters. After the best performing model was identified, it was then deployed in the test dataset containing the remaining 20% of participants (*n* = 1307). The accuracy of the model in terms of assigning participants to *effect* and *no effect* groups in the test dataset is shown in Figure 2. Overall, a classifier accuracy of 76% and an AUC of 0.84 was obtained, suggesting that the model was moderately accurate in assigning individuals to their respective groups.

**Figure 3:**
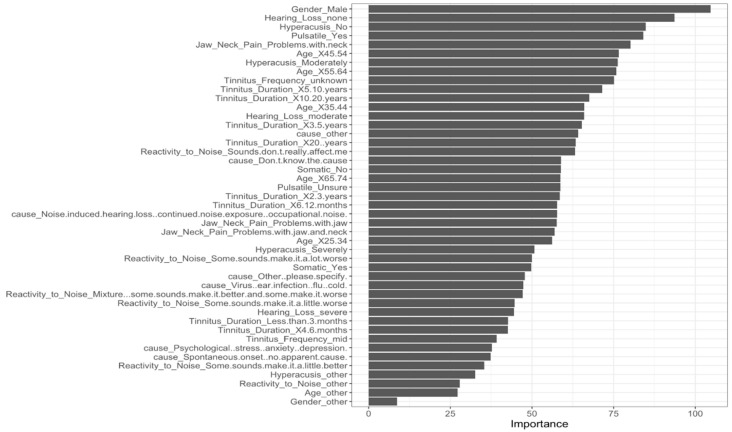
Variables most significantly affecting classification.

Figure 3 presents the participants’ demographic, comorbidity, and tinnitus characteristic variables most heavily weighted by the classification algorithm developed for this study. Somewhat surprisingly, male gender was identified as the most important predictor for classifying participants, despite the essentially even distribution of males and females across both groupings (see Table 1). Of the 10 top predictors, 3 were related to participant demographics (i.e., age and gender), 2 were related to tinnitus characteristics (i.e., tinnitus duration and frequency), and 5 were related to comorbidities (i.e., hearing loss, pulsatile tinnitus, jaw and/or neck problems, and hyperacusis). This finding highlights the multifactorial nature of the relationship between nutrition and tinnitus, and is consistent with the difficulties encountered when attempting to establish evidence-based dietary recommendations for those with tinnitus. Further, it suggests a partial explanation for apparent contradictions between available reports, which have largely relied upon univariable analysis methods.

## 4. Discussion

Whereas the potential for nutrition to impact outcomes in most chronic conditions is widely accepted [15,16], the influence of dietary choices on tinnitus remains inadequately understood at both group and individual levels. The present study reports results of a large, online survey investigating the perceived association between 10 dietary items suspected to influence tinnitus and tinnitus severity, with the aim of extending existing knowledge through the inclusion of a heterogeneous sample and utilization of modern data analysis techniques. Taken together, the results suggest that, while caffeine, alcohol, and salt were most likely to affect tinnitus severity, they did so only for a relatively small proportion of study participants. Furthermore, though a classification algorithm was able to leverage participant demographic, comorbidity, and tinnitus characteristics to identify those individuals most likely to benefit from dietary modification above chance levels, further efforts will be required to achieve significant clinical utility.

The potential benefit of restricting dietary intake of caffeine, salt, and alcohol is commonly discussed within tinnitus self-help groups and online forums, as well as within the scientific literature [39,59,60,61,62]. It is perhaps unsurprising, then, that respondents to the present survey reported these items as being among those items most likely to cause a worsening of tinnitus symptoms. The mechanisms through which caffeine, salt, and alcohol are proposed to worsen tinnitus include exacerbating effects on blood pressure, vasoconstriction within the cochlea, alteration of endolymph composition, and stimulatory effects on the central nervous system, which might interfere with central auditory processing [59,63,64]. Despite the widespread and persistent nature of these theories, high-quality studies have failed to identify the predicted effects. For example, in a recent randomized, triple-placebo-controlled clinical trial investigating the effect of caffeine on tinnitus severity, Ledesma et al. [60] could identify no acute effect of a 300 mg dose of caffeine on subjective ratings of tinnitus severity. Similarly, in a placebo-controlled crossover trial lasting 30 days, Claire et al. [65] could identify no evidence justifying caffeine abstinence as a treatment for preexisting tinnitus. In regard to alcohol, Stephens [61] reported an overwhelmingly detrimental effect of consumption on tinnitus level, with fully 84% of participants responding that tinnitus level increased with alcohol consumption. Somewhat counterintuitively, however, symptom severity did not appear related to either the type or amount of alcohol consumed. In partial contrast to this finding, a recent systematic review incorporating results of 11 studies failed to identify a definitive link between alcohol consumption and the likelihood of developing tinnitus. However, as evidenced by the results reported by Aljuaid et al. [39] in reference to caffeine consumption, the impact of nutrition on the development of tinnitus is not necessarily generalizable to preexisting tinnitus. Finally, in a Cochrane Library systematic review investigating the effectiveness of restricting either salt, caffeine, or alcohol for the treatment of Meniere’s disease, Hussain, Murdin [66] reported the absence of any high-quality, randomized, and controlled trials. Thus, the authors concluded that available data neither support nor refute dietary modification as a treatment for this common disorder. While these and similar studies do not provide conclusive evidence that caffeine, salt, and alcohol intake could not be responsible for increased tinnitus severity in certain individual ears, they do suggest that general, non-individualized recommendations should be avoided.

Significant inter-individual variability is evident in every available study assessing the effect of nutrition on tinnitus. In the present study, the overwhelming majority of respondents reported no effect of any dietary item on their tinnitus perception, which speaks against dietary modification being considered a primary treatment modality for people with chronic tinnitus in general. This finding is broadly consistent with previous work demonstrating the questionable effectiveness of various dietary restrictions on tinnitus when considered at the group level [59,67]. Despite this, it remains true that certain individuals did benefit from dietary modification. Thus, the clinical task becomes identifying those individuals *a priori*. The classification algorithm developed in this study performed above chance levels in terms of grouping individuals according to whether or not a dietary item might at all affect their tinnitus. It is possible that, by increasing the breadth and resolution of information provided to such an algorithm, prediction accuracy could be enhanced significantly. For example, by combining data collected by smartwatch-based heath tracker applications, calorie and food tracker applications, and ecological momentary assessment applications, a much more individualized assessment could be performed, potentially enhancing an algorithm’s ability to predict optimal nutrition-based tinnitus treatment strategies.

### 4.1. Future Work

This study’s findings are limited in terms of generalizability in several areas, each of which represents a direction for future research. First, the effect of dietary items on tinnitus was assessed via subjective, retrospective self-report. Thus, the potential for personal bias or imperfect memory to affect results cannot be excluded. Second, as data was gathered via anonymous survey, the completeness and accuracy of information on participant comorbidities cannot be guaranteed. Third, while the 10 dietary items selected for inclusion in the survey are commonly suggested to affect tinnitus, it is possible that certain non-included items would have been more effective in altering participants’ tinnitus perception. Further, it is likely that insufficiently clear item descriptions, such as “Junk Food”, “MSG”, and “Spicy Food” were interpreted differently by various participants. Fourth, while an online mode of survey distribution was selected in an effort to include the most heterogeneous and generalizable participant cohort as possible, it is possible that TinnitusTrack forum users might differ from the broader population in regard to degree of motivation, technological sophistication, or some other pertinent factor. Future studies incorporating online distribution models should take steps to ensure inclusion of multiple, distinct participant groups.

### 4.2. Conclusions

In recent years, demand has increased for nutrition-focused treatment options for chronic tinnitus. Results of the present study suggest that, while intake of caffeine, alcohol, and salt were most likely to influence tinnitus severity, they did so only for a relatively small proportion of participants. Furthermore, though a classification algorithm was able to leverage participant demographic, comorbidity, and tinnitus characteristics to identify those individuals most likely to benefit from dietary modification satisfactorily, further efforts are required to achieve significant clinical utility. Future research might focus on combining data across multiple mHealth applications, as doing so has the potential to allow for the generation of more accurate, personalized recommendations for the dietary management of chronic tinnitus.

## Figures and Tables

**Table 1:** Participant demographics, reported comorbidities, and tinnitus characteristics. Data were stratified based on the reported effect of dietary items on tinnitus. All *p*-values were corrected for multiple comparisons using the Holm method. Effect sizes were calculated using Cramer’s V.

	Effect (*n* = 1621)	No Effect (*n* = 3396)	Adjusted *p*-Values	Effect Size
*Age*			<0.001	0.09
Under 18 years of age	13 (0.8%)	39 (1.1%)		
18–24	99 (6.1%)	171 (5.0%)		
25–34	211 (13.0%)	360 (10.6%)		
35–44	256 (15.8%)	423 (12.5%)		
45–54	353 (21.8%)	694 (20.4%)		
55–64	462 (28.5%)	1046 (30.8%)		
65–74	198 (12.2%)	558 (16.4%)		
75+	27 (1.7%)	91 (2.7%)		
Prefer not to say	2 (0.1%)	14 (0.4%)		
*Gender*			1	0.02
Female	688 (42.4%)	1444 (42.5%)		
Male	925 (57.1%)	1927 (56.7%)		
Transgender	6 (0.4%)	11 (0.3%)		
Prefer not to say	2 (0.1%)	14 (0.4%)		
*Duration of tinnitus*			0.01	0.07
Less than 3 months	81 (5.0%)	205 (6.0%)		
4–6 months	75 (4.6%)	182 (5.4%)		
6–12 months	163 (10.1%)	288 (8.5%)		
1–2 years	245 (15.1%)	414 (12.2%)		
2–3 years	183 (11.3%)	346 (10.2%)		
3–5 years	194 (12.0%)	395 (11.6%)		
5–10 years	250 (15.4%)	571 (16.8%)		
10–20 years	225 (13.9%)	463 (13.6%)		
20+ years	205 (12.6%)	532 (15.7%)		
*Effect of noise on tinnitus*			<0.001	0.18
I don’t know	162 (10.0%)	490 (14.4%)		
Both improve and worsen tinnitus	437 (27.0%)	625 (18.4%)		
Some sounds make it a little better	102 (6.3%)	260 (7.7%)		
Some sounds make it a little worse	204 (12.6%)	365 (10.7%)		
Some sounds make it a lot better	72 (4.4%)	127 (3.7%)		
Some sounds make it a lot worse	400 (24.7%)	611 (18.0%)		
Sounds don’t really affect me	244 (15.1%)	918 (27.0%)		
*Hyperacusis*			<0.001	0.10
Don’t know	48 (3.0%)	152 (4.5%)		
Mildly	472 (29.1%)	829 (24.4%)		
Moderately	430 (26.5%)	737 (21.7%)		
No	509 (31.4%)	1384 (40.8%)		
Severely	162 (10.0%)	294 (8.7%)		
*Pulsatile*			1	0.02
Yes	299 (18.4%)	590 (17.4%)		
No	1165 (71.9%)	2486 (73.2%)		
Unsure	157 (9.7%)	320 (9.4%)		
*Somatic tinnitus*			<0.001	0.09
Yes	628 (38.7%)	1019 (30.0%)		
No	857 (52.9%)	2093 (61.6%)		
Don’t know	136 (8.4%)	284 (8.4%)		
*Jaw/neck problems*			<0.001	0.08
Problems with jaw	160 (9.9%)	277 (8.2%)		
Problems with neck	284 (17.5%)	601 (17.7%)		
Problems with jaw and neck	249 (15.4%)	350 (10.3%)		
None	928 (57.2%)	2168 (63.8%)		
*Hearing loss*			1	0.02
Mild	702 (43.3%)	1437 (42.3%)		
Moderate	234 (14.4%)	508 (15.0%)		
Severe	103 (6.4%)	196 (5.8%)		
None known of	582 (35.9%)	1255 (37.0%)		
*Tinnitus frequency*			0.001	0.06
high	300 (18.5%)	531 (15.6%)		
mid	107 (6.6%)	157 (4.6%)		
unknown	1214 (74.9%)	2708 (79.7%)		

## Data Availability

The data reported in this manuscript may be made available upon reasonable request.

## Supplementary Materials

The following supporting information can be downloaded at: https://www.mdpi.com/article/10.3390/nu14245356/s1, Figure S1: Reported associations of 10 dietary items with tinnitus.

## References

[1] De Ridder D., Schlee W., Vanneste S., Londero A., Weisz N., Kleinjung T., Shekhawat G.S., Elgoyhen A.B., Song J.J., Andersson G. (2021). Tinnitus and tinnitus disorder: Theoretical and operational definitions (an international multidisciplinary proposal). Prog. Brain Res..

[2] McCormack A., Edmondson-Jones M., Somerset S., Hall D. (2016). A systematic review of the reporting of tinnitus prevalence and severity. Hear. Res..

[3] Biswas R., Lugo A., Akeroyd M.A., Schlee W., Gallus S., Hall D.A. (2022). Tinnitus prevalence in Europe: A multi-country cross-sectional population study. Lancet Reg. Health Eur..

[4] Jarach C.M., Lugo A., Scala M., van den Brandt P.A., Cederroth C.R., Odone A., Garavello W., Schlee W., Langguth B., Gallus S. (2022). Global Prevalence and Incidence of Tinnitus: A Systematic Review and Meta-analysis. JAMA Neurol..

[5] Davis A., El Rafaie A. (2000). Epidemiology of Tinnitus.

[6] Henry J.A., Dennis K.C., Schechter M.A. (2005). General review of tinnitus. J. Speech Lang. Hear. Res..

[7] Henry J.A., Roberts L.E., Caspary D.M., Theodoroff S.M., Salvi R.J. (2014). Underlying mechanisms of tinnitus: Review and clinical implications. J. Am. Acad. Audiol..

[8] Mulders W.H., Ding D., Salvi R., Robertson D. (2011). Relationship between auditory thresholds, central spontaneous activity, and hair cell loss after acoustic trauma. J. Comp. Neurol..

[9] Salvi R.J., Wang J., Ding D. (2000). Auditory plasticity and hyperactivity following cochlear damage. Hear. Res..

[10] Bhatt J.M., Lin H.W., Bhattacharyya N. (2016). Prevalence, Severity, Exposures, and Treatment Patterns of Tinnitus in the United States. JAMA Otolaryngol. Head Neck Surg..

[11] Simoes J., Neff P., Schoisswohl S., Bulla J., Schecklmann M., Harrison S., Vesala M., Langguth B., Schlee W. (2019). Toward Personalized Tinnitus Treatment: An Exploratory Study Based on Internet Crowdsensing. Front. Public Health.

[12] Luetzenberg F.S., Babu S., Seidman M.D. (2020). Alternative Treatments of Tinnitus: Alternative Medicine. Otolaryngol. Clin. North Am..

[13] Hall D.A., Mohamad N., Firkins L., Fenton M., Stockdale D. (2013). Identifying and prioritizing unmet research questions for people with tinnitus: The James Lind Alliance Tinnitus Priority Setting Partnership. Clin. Investig..

[14] Tapsell L.C., Neale E.P., Satija A., Hu F.B. (2016). Foods, Nutrients, and Dietary Patterns: Interconnections and Implications for Dietary Guidelines. Adv. Nutr. (Bethesda, Md.).

[15] Ojo O. (2019). Nutrition and Chronic Conditions. Nutrients.

[16] Allison R.L. (2017). Back to Basics: The Effect of Healthy Diet and Exercise on Chronic Disease Management. S. D. Med..

[17] Wang L.L., Wang Q., Hong Y., Ojo O., Jiang Q., Hou Y.Y., Huang Y.H., Wang X.H. (2018). The Effect of Low-Carbohydrate Diet on Glycemic Control in Patients with Type 2 Diabetes Mellitus. Nutrients.

[18] World Heath Organization (2016). Global Report on Diabetes.

[19] Miller V., Micha R., Choi E., Karageorgou D., Webb P., Mozaffarian D. (2022). Evaluation of the Quality of Evidence of the Association of Foods and Nutrients With Cardiovascular Disease and Diabetes: A Systematic Review. JAMA Netw. Open.

[20] Petimar J., Park Y.M., Smith-Warner S.A., Fung T.T., Sandler D.P. (2019). Dietary index scores and invasive breast cancer risk among women with a family history of breast cancer. Am. J. Clin. Nutr..

[21] Petimar J., Smith-Warner S.A., Fung T.T., Rosner B., Chan A.T., Hu F.B., Giovannucci E.L., Tabung F.K. (2018). Recommendation-based dietary indexes and risk of colorectal cancer in the Nurses’ Health Study and Health Professionals Follow-up Study. Am. J. Clin. Nutr..

[22] Puzzono M., Mannucci A., Grannò S., Zuppardo R.A., Galli A., Danese S., Cavestro G.M. (2021). The Role of Diet and Lifestyle in Early-Onset Colorectal Cancer: A Systematic Review. Cancers.

[23] Potter J., Brown L., Williams R.L., Byles J., Collins C.E. (2016). Diet Quality and Cancer Outcomes in Adults: A Systematic Review of Epidemiological Studies. Int. J. Mol. Sci..

[24] Movassagh E.Z., Vatanparast H. (2017). Current Evidence on the Association of Dietary Patterns and Bone Health: A Scoping Review. Adv. Nutr..

[25] Melaku Y.A., Gill T.K., Adams R., Shi Z. (2016). Association between dietary patterns and low bone mineral density among adults aged 50 years and above: Findings from the North West Adelaide Health Study (NWAHS). Br. J. Nutr..

[26] Yamagishi K., Maruyama K., Ikeda A., Nagao M., Noda H., Umesawa M., Hayama-Terada M., Muraki I., Okada C., Tanaka M. (2022). Dietary fiber intake and risk of incident disabling dementia: The Circulatory Risk in Communities Study. Nutr. Neurosci..

[27] Frausto D.M., Forsyth C.B., Keshavarzian A., Voigt R.M. (2021). Dietary Regulation of Gut-Brain Axis in Alzheimer’s Disease: Importance of Microbiota Metabolites. Front. Neurosci..

[28] Spankovich C., Le Prell C.G. (2019). The role of diet in vulnerability to noise-induced cochlear injury and hearing loss. J. Acoust. Soc. Am..

[29] Dawes P., Cruickshanks K.J., Marsden A., Moore D.R., Munro K.J. (2020). Relationship Between Diet, Tinnitus, and Hearing Difficulties. Ear Hear..

[30] Huang Q., Jin Y., Reed N.S., Ma Y., Power M.C., Talegawkar S.A. (2020). Diet quality and hearing loss among middle-older aged adults in the USA: Findings from National Health and Nutrition Examination Survey. Public Health Nutr..

[31] Puga A.M., Pajares M.A., Varela-Moreiras G., Partearroyo T. (2018). Interplay between Nutrition and Hearing Loss: State of Art. Nutrients.

[32] Curhan S.G., Wang M., Eavey R.D., Stampfer M.J., Curhan G.C. (2018). Adherence to Healthful Dietary Patterns Is Associated with Lower Risk of Hearing Loss in Women. J. Nutr..

[33] Curhan S.G., Halpin C., Wang M., Eavey R.D., Curhan G.C. (2020). Prospective Study of Dietary Patterns and Hearing Threshold Elevation. Am. J. Epidemiol..

[34] Spankovich C., Bishop C., Johnson M.F., Elkins A., Su D., Lobarinas E., Le Prell C.G. (2017). Relationship between dietary quality, tinnitus and hearing level: Data from the national health and nutrition examination survey, 1999-2002. Int. J. Audiol..

[35] Lee D.Y., Kim Y.H. (2018). Relationship Between Diet and Tinnitus: Korea National Health and Nutrition Examination Survey. Clin. Exp. Otorhinolaryngol..

[36] Spankovich C., Le Prell C.G. (2013). Healthy diets, healthy hearing: National Health and Nutrition Examination Survey, 1999–2002. Int. J. Audiol..

[37] Spankovich C. (2015). The role of nutrition in healthy hearing: Human evidence. Free Radicals in ENT Pathology.

[38] Tang D., Tran Y., Shekhawat G.S., Burlutsky G., Mitchell P., Gopinath B. (2021). Dietary Fibre Intake and the 10-Year Incidence of Tinnitus in Older Adults. Nutrients.

[39] Aljuaid S.M., Mirza A.A., Habib L.A., AlHarthi L.A., Alansari B.M., AlQahtani B.G., Althobaiti Y.A. (2021). Does Caffeine Intake Increase the Incidence of Tinnitus? A Systematic Review. Int. Arch. Otorhinolaryngol..

[40] Zhao H.-H., Ma Z.-C., Sun Y.-N. (2020). A Risk Assessment Approach of Hypertension Based on Mobile Crowd Sensing. J. Inf. Sci. Eng..

[41] Gardašević G., Katzis K., Bajić D., Berbakov L. (2020). Emerging Wireless Sensor Networks and Internet of Things Technologies-Foundations of Smart Healthcare. Sensors.

[42] Kraft R., Schlee W., Stach M., Reichert M., Langguth B., Baumeister H., Probst T., Hannemann R., Pryss R. (2020). Combining Mobile Crowdsensing and Ecological Momentary Assessments in the Healthcare Domain. Front. Neurosci..

[43] Simsek M., Kantarci B. (2020). Artificial Intelligence-Empowered Mobilization of Assessments in COVID-19-like Pandemics: A Case Study for Early Flattening of the Curve. Int. J. Environ. Res. Public Health.

[44] Pryss R., Schobel J., Hoppenstedt B., Spiliopoulou M., Langguth B., Probst T., Schlee W., Reichert M., Kurthen I., Giroud N. Ecological Momentary Assessment based Differences between Android and iOS Users of the TrackYourHearing mHealth Crowdsensing Platform. Proceedings of the Annual International Conference of the IEEE Engineering in Medicine and Biology Society. IEEE Engineering in Medicine and Biology Society Annual International Conference.

[45] Vogel C., Schobel J., Schlee W., Engelke M., Pryss R. UNITI Mobile-EMI-Apps for a Large-Scale European Study on Tinnitus. Proceedings of the Annual International Conference of the IEEE Engineering in Medicine and Biology Society IEEE Engineering in Medicine and Biology Society Annual International Conference.

[46] Probst T., Pryss R.C., Langguth B., Spiliopoulou M., Landgrebe M., Vesala M., Harrison S., Schobel J., Reichert M., Stach M. (2017). Outpatient Tinnitus Clinic, Self-Help Web Platform, or Mobile Application to Recruit Tinnitus Study Samples?. Front. Aging Neurosci..

[47] Tang D., Tran Y., Lewis J.R., Bondonno N.P., Bondonno C.P., Hodgson J.M., Domingo D., McAlpine D., Burlutsky G., Mitchell P. (2022). Associations between intake of dietary flavonoids and the 10-year incidence of tinnitus in older adults. Eur. J. Nutr..

[48] Longadge R., Dongre S. (2013). Class imbalance problem in data mining review. arXiv.

[49] Malarvizhi R., Thanamani A.S. (2012). K-nearest neighbor in missing data imputation. Int. J. Eng. Res. Dev..

[50] Kuhn M., Johnson K. (2019). Feature Engineering and Selection: A Practical Approach for Predictive Models.

[51] Chawla N.V., Bowyer K.W., Hall L.O., Kegelmeyer W.P. (2002). SMOTE: Synthetic minority over-sampling technique. J. Artif. Intell. Res..

[52] Breiman L. (2001). Random Forests. Mach. Learn..

[53] Greenwell B.M., Boehmke B.C., Gray B. (2020). Variable Importance Plots-An Introduction to the vip Package. R J..

[54] Holm S. (1979). A Simple Sequentially Rejective Multiple Test Procedure. Scand. J. Stat..

[55] Ellis P.D. (2010). The Essential Guide to Effect Sizes: Statistical Power, Meta-Analysis, and the Interpretation of Research Results.

[56] R Core Team (2018). R: A Language and Environment for Statistical Computing.

[57] Wickham H., Averick M., Bryan J., Chang W., McGowan L.D.A., François R., Grolemund G., Hayes A., Henry L., Hester J. (2019). Welcome to the Tidyverse. J. Open Source Softw..

[58] Kuhn M., Wickham H. (2020). Tidymodels: A Collection of Packages for Modeling and Machine Learning Using Tidyverse Principles. Boston, MA, USA. https://tidymodels.org.

[59] Hofmeister M. (2019). Do dietary factors significantly influence tinnitus?. Aust. J. Gen. Pract..

[60] Ledesma A.L.L., Leite Rodrigues D., Monteiro de Castro Silva I., Oliveira C.A., Bahmad F. (2021). The effect of caffeine on tinnitus: Randomized triple-blind placebo-controlled clinical trial. PLoS ONE.

[61] Stephens D. (1999). Detrimental effects of alcohol on tinnitus. Clin. Otolaryngol. Allied Sci..

[62] Pugh R., Budd R.J., Stephens S.D. (1995). Patients’ reports of the effect of alcohol on tinnitus. Br. J. Audiol..

[63] Mazzoli M., Møller A.R., Langguth B., De Ridder D., Kleinjung T. (2011). Complementary Tinnitus Therapies. Textbook of Tinnitus.

[64] Trinidade A., Robinson T., Phillips J.S. (2014). The role of caffeine in otorhinolaryngology: Guilty as charged?. Eur. Arch. Otohinolaryngol..

[65] Claire L.S., Stothart G., McKenna L., Rogers P.J. (2010). Caffeine abstinence: An ineffective and potentially distressing tinnitus therapy. Int. J. Audiol..

[66] Hussain K., Murdin L., Schilder A.G.M. (2018). Restriction of salt, caffeine and alcohol intake for the treatment of Ménière’s disease or syndrome. Cochrane Database Syst. Rev..

[67] Biswas R., Lugo A., Genitsaridi E., Trpchevska N., Akeroyd M.A., Cederroth C.R., Liu X., Schlee W., Garavello W., Gallus S., Langguth B., Kleinjung T., Ridder D.D., Schlee W., Vanneste S. (2021). Chapter 1—Modifiable lifestyle-related risk factors for tinnitus in the general population: An overview of smoking, alcohol, body mass index and caffeine intake. Progress in Brain Research.

