# Supplementary figures and images for: The Influence of Diet on Tinnitus Severity: Results of a Large-Scale, Online Survey
Source: Nutrients. 2022 Dec 16;14(24):5356. doi: 10.3390/nu14245356 (PMC9784733; doi:10.3390/nu14245356)

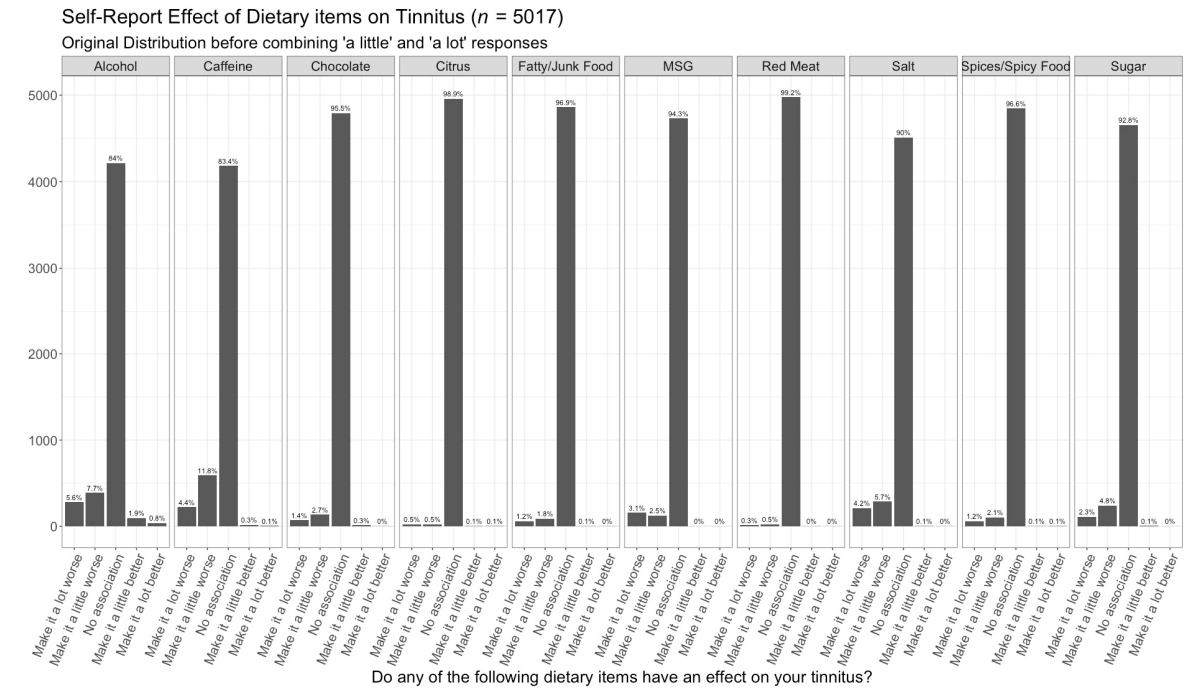

Figure S1: Reported associations of 10 dietary items with tinnitus.

Supplement: Supplementary file 1 [file nutrients-14-05356-s001.zip › nutrients-2005300-supplementary.pdf]
